# Severe cytomegalovirus infection in apparently immunocompetent patients: a systematic review

**DOI:** 10.1186/1743-422X-5-47

**Published:** 2008-03-27

**Authors:** Petros I Rafailidis, Eleni G Mourtzoukou, Ioannis C Varbobitis, Matthew E Falagas

**Affiliations:** 1 Alfa Institute of Biomedical Sciences (AIBS), Athens, Greece; 2 Department of Medicine, Henry Dunant Hospital, Athens, Greece; 3 Department of Medicine, Tufts University School of Medicine, Boston, Massachusetts, USA

## Abstract

**Background:**

The morbidity and mortality associated with cytomegalovirus (CMV) infection in immunocompromised patients (especially in HIV-infected patients and transplant recipients), as well as with congenital CMV infection are well known. In contrast, relatively little attention has been paid to the morbidity and mortality that CMV infection may cause in immunocompetent patients.

**Methods:**

We reviewed the evidence associated with severe manifestations of CMV infection in apparently immunocompetent patients and the potential role of antiviral treatment for these infections. We searched in PubMed, Scopus, and the Cochrane Library for the period of 1950–2007 to identify relevant articles.

**Results:**

We retrieved 89 articles reporting on severe CMV infection in 290 immunocompetent adults. Among these reports, the gastrointestinal tract (colitis) and the central nervous system (meningitis, encephalitis, transverse myelitis) were the most frequent sites of severe CMV infection. Manifestations from other organ-systems included haematological disorders (haemolytic anaemia, thrombocytopenia), thrombosis of the venous or arterial vascular system, ocular involvement (uveitis), and lung disease (pneumonitis). The clinical practice reported in the literature has been to prescribe antiviral treatment for the most severe manifestations of monophasic meningoencephalitis (seizures and coma), ocular involvement, and lung involvement due to CMV.

**Conclusion:**

Severe life-threatening complications of CMV infection in immunocompetent patients may not be as rare as previously thought.

## Introduction

Cytomegalovirus (CMV) can cause severe disease in immunocompromised patients, either via reactivation of latent CMV infection or via acquisition of primary CMV infection. Clinical syndromes that may be observed in this setting include encephalitis, pneumonitis, hepatitis, uveitis, retinitis, colitis, and graft rejection. Furthermore, CMV infection affecting the human embryo, a host with immature immunologic responses, is often associated with serious complications, such as microcephaly, mental retardation, spastic paralysis, hepatosplenomegaly, anaemia, thrombocytopenia, deafness, and optic nerve atrophy leading to blindness.

To the contrary, in immunocompetent patients, primary CMV infection typically runs an undifferentiated viral syndrome, or is manifested by a mononucleosis-like syndrome. Infections in the immunocompetent and immunosupressed are not rare; seroprevalence for CMV worldwide ranges from ~60%–100% [1]. Symptomatic CMV infection in non-immunocompromised hosts has traditionally been considered to have a benign, self-limited course. However, in the medical literature there are a considerable number of reports of severe clinical manifestations of CMV infection in immunocompetent patients.

An issue that has not been comprehensively resolved is the potential role of specific antiviral treatment for immunocompetent patients with pronounced clinical manifestations related to CMV infection. Although current opinion is that CMV infection in immunocompetent patients does not require treatment, the risks and benefits of specific antiviral treatment for severely ill patients are not adequately addressed. In this context, we sought to review the biomedical literature for reports regarding severe CMV infections in immunocompetent patients, and to evaluate, on the basis of existing evidence, the role of antiviral treatment, if any, for these patients.

## Methods

### Literature search

We performed a systematic review of the literature regarding serious manifestations of CMV infection in apparently immunocompetent individuals. Two reviewers (PIR and EGM) independently searched PubMed, Scopus, and the Cochrane Library for relevant articles published between 1950 and 2007. Search terms applied were "CMV", "cytomegalovirus", "severe", "immunocompetent", "fulminant", and "fatal", in various combinations. The reference lists of relevant articles retrieved by the searches were also reviewed. Reports included in our review were limited to those written in English, German, or French.

### Study selection criteria

We included any study that reported severe CMV infection in immunocompetent patients. We defined as severe any CMV infection for which the patient was hospitalized and/or the infection was deemed to be of a life-threatening degree. The various types of CMV infections were grouped with regard to the afflicted body system or site, as defined by the authors of the original reports, into infections of: the gastrointestinal tract; the central nervous system; lungs; eyes; or skin. In addition, categories of CMV infections characterized as severe included any CMV infection causing vascular thrombosis; weight loss of over 10 kg; a vasculitic rash; perineal ulcers; or an eruption consisting of numerous vesicular or pustular lesions, throughout the body; any CMV infection causing liver involvement of a degree to necessitate hospitalisation, or a liver biopsy, or accompanied by at least a five-fold rise of alanine transaminase serum value or jaundice. Immunological competence was defined by the absence of: a congenital or acquired immunodeficiency syndrome; a history of allogeneic transplantation (except for corneal transplantation); or immunosuppressive treatment (including antineoplastic chemotherapy, and long-term glucocorticosteroid therapy). We excluded cases of congenital CMV infection, patients with inflammatory bowel disease that had received systemic or topical steroids in the month preceding the CMV infection, and, patients with clinical syndromes that are attributed to aberrant immunological responses triggered by the presence of CMV, rather than to tissue damage related to active replication of the virus (such as the Guillain-Barré syndrome).

The diagnosis of CMV infection for this review required at least one of the following laboratory methods: serology, specific intrathecal antibody production, virus isolation, direct detection of CMV pp65 antigen in blood, CMV culture, biopsy, positive specific immunohistochemical staining, polymerase chain reaction (PCR) assay (mainly quantitative results examined together with clinical findings as false -positive results are a possibility), confocal microscopy of the eyes (to detect the "owl's eye" morphology in the corneal endothelium), or in situ hybridization. Serological studies indicating an acute CMV infection included the presence of positive IgM anti-CMV antibodies, or of a significant increase in the titre of IgG anti-CMV antibodies in paired samples obtained during the infection.

Three reviewers (PIR, EGM, ICV) independently assessed all retrieved articles, on the basis of title and abstract, for the purpose of determining eligibility for inclusion in the systematic review. Any differences in the extracted data between the three reviewers were resolved in meetings of all authors.

### Definition of infection outcomes

Cure was defined as the complete resolution of symptoms and signs attributed to the CMV infection, in conjunction with normalization of any related laboratory abnormalities. Improvement was defined as partial resolution of symptoms and signs attributed to the CMV infection, with clinical stability of any associated residual organ dysfunction. Failure was defined as lack of improvement, or deterioration, or death attributed to the CMV infection. Death due to other concomitant illness was not taken into account in determining infection outcome.

## Results

The literature search yielded 273 articles with potential relevance to our study. After screening these articles on the basis of title and abstract, we excluded 184 of them, primarily because they did not focus on severe CMV infections in immunocompetent patients. Thus, 89 different studies, reporting on 290 patients, were identified for our review [[2-8], Additional file 1]. The table 'review of case reports of severe CMV infections in immunocompetent patients' (see Additional file 1) summarizes data extracted from studies that were not included in previous relevant reviews, and thus they were first identified in this review. In Table 1 we summarize the findings of previous reviews relevant to our study.

**Table 1:** Synopsis of data from previous reviews on severe CMV infections and in immunocompetent patients.

**Reference Year of publication**	**Number of patients**	**Site involved**	**Patient demographics**	**Comorbidity**	**Antiviral treatment**	**Outcome**
[2]/2007	11	Portal vein thrombosis	55% M/MA 34 y and one infant 4 m	Known thromboembolic risk factors in 64%	ND	Complete thrombus resolution in 73% of patients 2/11 received antiviral treatment
[3]/2005	44	Colitis	61.5% M/61.1(25–71)	18 IM (8 with renal failure, 6 with DM, 2 pregnant), 18 with NM and 10 NC	18.8% in the IM 44% in the NM group 40% in the NC group	MR and SR: 56.3% and 18.8% in the IM, 22% and 33% in the NM group, 10% and 50% in the NC group
[4]/2003	4	Vascular thrombosis and pulmonary embolism	40% M/29–38 y and one neonate	One patient APA, and another FVLH	Ganciclovir one patient, none another ND for 3 patients	Good for personal 2 patients (1 of them received treatment) ND for 3
[5]/2001	15	Colitis	MA 63+/-20 y	ND	34% received AT	31% in the AT group died 36% of those that did not receive AT died**
[6]/2000	19	Meningoencephalitis [16 monophasic (2 of them had multiorgan involvement)] and 3 paroxysmal]	17–78 y	ND	12/16 with monophasic received treatment (8 acyclovir, 3 ganciclovir,1 adenine-arabinoside) None with paroxysmal received treatment	Monophasic without treatment: 4/5 had CR while 1/5 PR With treatment: 13/16 CR, 1/16 PR, 2/16 deaths Paroxysmal: All CR
[7]/1997	27	Hepatitis (15), Nervous system (9), Pneumonitis (2), colitis (1)	44% males, 14–73 y	3 pregnant women	5 ganciclovir, 1 acyclovir, 2 vidarabine, 19 none	1/5 of those who received ganciclovir died, 13/22 of the remaining died
[8]/1985	62	Myocarditis (10), pneumonia (8), encephalitis (7), gastrointestinal infection (8), granulomatous hepatitis (7), haemolytic anaemia (5), icteric hepatitis (3), conjunctivitis (3) severe thrombocytopenia (2), pericarditis (2), meningitis (2), VIII cranial nerve palsy (1), uveitis (1), chorioretinitis (1), rash (2)	ND for the majority 39.8 years old (for patients with granulomatous hepatitis, uveitis, chorioretinitis, gastrointestinal)	ND	ND	All survived except for 3 who died (1 with pericarditis and 2 with gastrointestinal infection) Relapses in one patient with uveitis and persistence in 1 with chorioretinitis

**Abbreviations **PVT: portal vein thrombosis, MA: mean age, y: years, m: months, NM: non immune-modulating conditions, NC: no comorbidities, MR: mortality rate, SR: spontaneous remission rate, IBD: inflammatory bowel disease, APA: antiphospholipid antibodies, FVLH: factor V Leiden heterozygous mutation, ND: no data, AT: antiviral treatment, CR: complete recovery, PR: partial recovery, CMV: cytomegalovirus.

**: patients with more severe infections were administered AT.

^#^:Total number of reports by Karakozis is 38. However 23 patients reviewed by Karakozis and Galiatsatos are the same, and thus deducted from the Karakozis study.

The gastrointestinal tract (GIT) was the primary site of involvement by the CMV infection in 91 patients (32 identified in this review plus 59 included in previous reviews, performed by Galliatsatos et al [3]; and Karakozis et al [5]). As identified by the diagnostic tests performed in the respective studies, CMV infection of the GIT was classified as gastroenteritis, duodenitis, ileitis, colitis, proctitis, or exacerbation of inflammatory bowel disease. Concurrent manifestations of CMV disease regarding other organ systems than the GIT, e.g. haemolytic anaemia or bleeding diathesis, was identified in a limited number of cases. The symptoms observed in patients with CMV involvement of the GIT included fever, diffuse abdominal pain, or pain located in the lower abdominal quadrants, anorexia, nausea, vomiting, weight loss, watery or bloody diarrhoea, haematochezia, and melaena. The signs recorded in the physical examination of these patients, included abdominal tenderness, or rebound tenderness and abnormal bowel sounds. The diagnostic investigations performed in these patients, included abdominal ultrasound, computed tomography of the abdomen, GIT endoscopy (gastroscopy, colonoscopy, sigmoidoscopy) with biopsy, and stool cultures. Fourteen out of the 32 cases identified received treatment with ganciclovir (in 3 cases administered in combination with valganciclovir), whereas 2 received valganciclovir alone, and 16 did not receive any antiviral therapy. Two patients died, one of which was receiving therapy.

Central nervous system (CNS) disorders constituted the second most frequent manifestations of CMV infection in immunocompetent patients. Specifically, patients with involvement of the CNS by cytomegalovirus presented with various combinations of the following symptoms and signs: fever, chills, fatigue, myalgia, motor deficits (localized weakness, paraplegia), sensory abnormalities (numbness, hypoaesthesia, paraesthesia, dysaesthesia, anaesthesia), disorientation, confusion, unilateral or bilateral visual loss, urinary retention, constipation, or coma.

Fifty-six immunocompetent patients with severe CNS CMV infection were identified (19 patients first reported herein plus 37 included in previous reviews performed by Devetag et al [6]; Eddleston et al [7]; and Cohen et al [8]). All of these patients presented with symptoms and signs of myelitis, encephalomyelitis, encephalitis, meningoencephalitis, meningitis, or meningoradiculopathy. Nine of the 19 patients that were first identified in this review received specific antiviral treatment (6 with ganciclovir, 2 with acyclovir, and 1 with valganciclovir), whereas 10 of these patients did not receive any specific antiviral therapy. None of the 19 patients died.

Twenty-five immunocompetent patients were identified as having haematological disorders caused by CMV infection. These disorders included: symptomatic thrombocytopenia, haemolytic anaemia, disseminated intravascular coagulation, myelodysplastic changes, pancytopenia, and splenic rupture. This group of patients presented with a diversity of symptoms, including fatigue, fever, abdominal or chest pain, headache, pain in the extremities, numbness of the hands, darkening of urine due to the presence of haemoglobin, epistaxis, easy bruising, purpura, increased incidence of infections, jaundice, and systolic ejection murmur.

Less frequent manifestations of severe CMV infections in immunocompetent patients included vascular thrombosis, ocular involvement, and pulmonary disease. Nineteen patients overall (4 presented herein and 15 reported previously by Squizzato et al. [2] and Abgueguen et al. [4]), presented with various types of blood vessel thrombosis, including thrombosis of the portal femoropopliteal veins and pulmonary embolism. A pro-coagulant status was identified in the 4 of this group of patients (2 were factor V Leiden heterozygotes, whereas 2 were receiving oral contraceptives). These patients presented with symptoms related to the specific site of vascular thrombosis, such as abdominal, chest, or pelvic pain, abdominal tenderness, dyspnoea and cyanosis, or hepatosplenomegaly. Some of these patients had non-specific symptoms, such as fever, myalgia, asthenia, chills, or cough.

The virus affected the eyes in 16 immunocompetent patients, who developed uveitis, retinitis, corneal endothelitis, or papillitis. Presenting symptoms and signs in these patients included loss or blurring of vision, as well as redness of the affected eyes. The diagnosis and the monitoring of response to therapy were performed by serum serology, slit-lamp examination, confocal microscopy, or by PCR of the aqueous humour, or of the vitreous. All but one of these patients received specific antiviral treatment. The outcome of the patient who did not receive antiviral treatment was not favourable. In addition, patients were treated with corticosteroids and topical therapy; timolol, atropine, cyclopentolate. Finally, patients underwent eye surgery when indicated.

Nine immunocompetent patients had lung involvement caused by CMV infection. Five of them developed pneumonia or interstitial pneumonitis. The symptoms and signs of CMV pneumonitis were non-specific, and included the following: chest pain, cough, haemoptysis, shortness of breath, fever, sweats, and cervical lymphadenopathy. The diagnosis in these patients was established with the use of serological tests, PCR in blood, PCR or immunohistochemistry in lung tissue (obtained through either transbronchial or open lung biopsy), as well as with analysis of bronchoalveolar lavage fluid. Four patients had a favourable outcome in response to specific antiviral therapy (3 of them received ganciclovir and one did not), while an additional patient, who received ganciclovir, died. The remaining 4 patients were diagnosed with septal capillary injury syndrome. Three of these patients received antiviral treatment and showed good response to treatment, while the remaining patient, who did not receive antiviral therapy, died.

## Discussion

While manifestations of CMV infection in immunocompromised hosts have been extensively reported in biomedical literature, those observed in immunocompetent patients have received comparatively little attention. The study we performed shows that severe life-threatening complications of CMV infection in immunocompetent patients may not be as rare as previously thought. Also, various considerations should be taken into account that may lead to underreporting of severe CMV infections. This may be, at least in part, due to the rather moderate accuracy of some routinely available diagnostic methods, such as serological tests. On the other hand, molecular diagnostic methods, such as PCR, that are not universally available, appear to be more sensitive than serological or virus isolation tests, while they are also more rapid [9]. As shown in our review severe CMV infections in immunocompetent patients can affect almost every system. In a decrescendo order of frequency, severe organ involvement in the herein reviewed reports included: the gastrointestinal tract (colitis), the central nervous system (meningitis, encephalitis, myelitis, nerve palsies, myeloradiculopathy), haematological manifestations (haemolytic anaemia and thrombocytopenia), the eye (uveitis, retinitis, liver (hepatitis), lung (pneumonitis) and thrombosis of the arterial and venous system (deep venous thrombosis, portal vein thrombosis, pulmonary embolism). Our data regarding severe CMV infections are in concordance with those of the literature examining cohorts with CMV infection or focusing on a severe organ involvement by the virus.

Relatively few cohort studies, of an appreciably large size, evaluating the incidence of severe CMV disease in immunocompetent patients, have been reported to date. In detail, among 116 immunocompetent adults (of 19–68 years of age) with acute CMV infection, that were studied retrospectively by Faucher et al., two (1.7%) developed severe complications (interstitial pneumonitis and encephalitis, respectively)[10]. In the above described study CMV infection was diagnosed on the basis of high initial titres of anti-CMV IgM antibodies in conjunction with a significant seroconversion in the IgG antibody titre during the course of illness. In another cohort of 115 patients with acute CMV infection (of 17–80 years of age), reported by Bonnet et al., 6 (5.2%) developed severe disease (3 of them presented with leg purpura, two with splenic haematoma and one with cutaneous vasculitis) [11]. The diagnosis of CMV infection was based on the initial presence of CMV IgM antibodies, in conjunction with subsequent seroconversion, or on the detection of CMV viraemia by blood cultures or of pp65 antigenaemia by immunofluorescent assay. In an additional cohort study reported by Wreghitt et al, of the 124 included immunocompetent patients (of 16–86 years of age) who were diagnosed with acute CMV infection, 28% suffered from respiratory symptoms, 24% had jaundice, and 3% presented with mental confusion, while 15% of the patients required hospitalisation [12]. The diagnosis was based on a high level of CMV IgM antibodies (>300 U/mL) coupled with the appearance of IgG specific antibodies 2–3 weeks after the onset of symptoms.

As is evident in our review, the site of the gastrointestinal tract most frequently affected by severe CMV disease in immunocompetent patients is the colon. These data are in concordance with those reported by Galliatsatos et al. [3] and Karakozis et al. [5]. The mortality rate however varies between the patients reviewed herein (6.2%) and those previously reported (32%). Among the latter group of patients there was a trend for higher mortality in patients over 55 years-old, and in patients with diseases affecting immune responses (diabetes mellitus, renal failure, pregnancy, and untreated non-haematological malignancy). In a pathologic study including an unselected group of 6323 patients, the rate of CMV identification in gastrointestinal mucosal biopsies was 9 per thousand. Specifically, characteristic CMV inclusion bodies were identified in 37 immunocompetent, and in 17 immunocompromised patients [13]. The most frequent gastrointestinal site of affliction of CMV disease in immunocompetent patients was the colon and rectum. Of note, characteristic CMV inclusions were present mainly in stromal and endothelial cells, rather than in macrophages.

It should also be mentioned that a special population afflicted by CMV disease consists of patients with pre-existing inflammatory bowel disease. It is suggested that cytokines like TNF-α and IFN-γ that are frequently elevated in patients with inflammatory bowel disease, can promote the reactivation of a latent CMV infection. This, in turn, is known to cause additional cytokine release, particularly of IL-6, a fact that may lead to exacerbation of the inflammatory bowel disease [14]. This sequence of events may be observed even in patients with inflammatory bowel disease not having recently received any steroid treatment. It is noteworthy that CMV colitis in patients with underlying inflammatory bowel disease has the potential to cause severe complications, such as toxic megacolon, colovesical fistula, perforation, and peritonitis.

The central nervous system (CNS) is not spared in CMV infections observed in immunocompetent patients as shown in our review. Our data are in concordance with those of Devetag et al who proposed that two types of CMV meningoencephalitis can be observed in immunocompetent patients: the paroxysmal type, which is characterized by focal neurological symptoms or signs, alternating side of neurological deficits, headache, symptoms lasting from minutes to hours, and generally a benign outcome, and, also, the monophasic type, which is characterized by more frequent occurrence of seizures, altered sensorium, symptoms lasting for days, and a less benign course [6]. In addition, advanced age portends a worse prognosis [6]. None of the patients in our review died. Among the 37 patients that were previously reviewed, 8 patients were treated with acyclovir and 1 died; 4 patients were treated with ganciclovir and 2 died; 3 patients were treated with vidarabine and survived and 22 patients did not receive any antiviral treatment and 4 of them died [6-8].

Thrombosis of the vascular system is another one of the potential manifestations of CMV infection in patients with normal immune responses. A variety of veins may be afflicted as shown in the patients reported in this review and in those previously reported by Squizzato et al. [2] and Abgueguen et al. [4]. No comprehensive interpretation of this association is yet available, but data strongly support a causal relationship, owed to the CMV intrinsic procoagulant properties. It appears that CMV directly invades vascular endothelial cells, causing membrane alterations that promote coagulation [2]. Another plausible explanation is that CMV infection can cause activation of vascular cells and the expression by the latter of adhesion molecules that react with platelets and leukocytes. In addition, CMV infection may be associated with over-expression of the platelet-derived growth factor, and of the transforming growth factor-β, which in turn causes vascular cell wall proliferation [15]. Finally, one of the immediate-early gene products of CMV, namely IE84, binds to p53 and inhibits its transcriptional activity, thus inhibiting p53-mediated apoptosis, and enhancing vascular smooth muscle cell proliferation [4].

It is suggested that the suppression of haematopoiesis that is associated with CMV infection may be due to direct inhibition by the virus of progenitor haematopoietic cell growth, as well as to stromal cell dysfunction, or to effects of inhibitory cytokines produced by CMV infected leukocytes. Furthermore, the detection of specific antibodies [16], and of other immunological abnormalities [17], in CMV-infected patients with haemolysis or myelodysplasia indicates a probable immune-mediated mechanism responsible for these manifestations.

Ocular CMV disease in immunocompromised individuals is known to involve more often the retina. Yet, as observed in our review, in apparently immunocompetent individuals, the retina and the anterior uvea are evenly affected. The higher rate of confinement of CMV infection in the anterior segment observed in the immunocompetent patients compared to immunocompromised ones may be attributed to the more effective immune response in the former group [18]. Still, anterior uveitis has been associated with long-term sequelae in some of the immunocompetent patients, such as iris atrophy and glaucoma. The development of these complications can be attributed to chronic irreversible trabecular alterations in patients having low-grade ocular inflammation for many years before the specific diagnosis of CMV infection was confirmed and antiviral treatment initiated. Of note, the introduction of anti-CMV therapy controlled secondary glaucoma in some of these patients [19].

The data identified in this review regarding the need for specific antiviral treatment in immunocompetent patients with severe CMV infection, are rather conflicting. No definitive conclusions can be drawn about the potential benefit of antiviral therapy for severe CMV disease based on these uncontrolled reports. The improvement observed in some of the treated patients may have been related to the typically self-limiting course of the disease, and thus cannot be attributed with certainty to a treatment effect. To evaluate the potential role of specific antiviral treatment for immunocompetent patients with severe CMV disease, randomized controlled trials are deemed necessary.

It should also be noted that any presumed benefit of specific antiviral treatment in severe cases of CMV infection, regarding immunocompetent patients, should be weighed against the potential toxicity of therapy. While adverse-effects associated with the administration of antiviral treatment against CMV were not reported in the reviewed cases, one should be aware that ganciclovir can cause myelosuppression, central nervous system disorders, hepatotoxicity, irreversible infertility (inhibition of spermatogenesis), or teratogenesis, whereas foscarnet can cause disturbances in mineral and electrolyte homeostasis, as well as nephrotoxicity. Additionally, long-term administration of these agents may lead to the emergence of resistant viral strains.

## Conclusion

In summary, severe life-threatening complications of CMV infection in immunocompetent patients may not be as rare as previously thought. No conclusive statements regarding the use of antiviral treatment can be made from the available data in the literature. However, as it is evident from this review, physicians generally tend to prescribe antiviral treatment for the most severe cases of monophasic CMV meningoencephalitis, as well as for patients with severe ocular involvement, and severe lung involvement, caused by the CMV infection. Randomized controlled trials are needed for a more conclusive answer on whether the use of antiviral treatment is indicated for immunocompetent patients suffering form severe CMV infection.

## Competing interests

The author(s) declare that they have no competing interests.

## Authors' contributions

PIR conceived the study and participated in its design and interpretation of data. EGM and ICV participated in the design, acquisition, analysis and interpretation of data. MEF participated in the design and coordination of the study and revised the manuscript critically for important intellectual content. All authors approved the final version of the manuscript.

## Supplementary Material

Additional file 1 Table. Review of case reports of severe CMV infections in immunocompetent patients. Data extracted from studies that were not included in previous relevant reviews, regarding cases of severe CMV infections in immunocompetent patients. Click here for file
