# Supplementary material for: Severe cytomegalovirus infection in apparently immunocompetent patients: a systematic review
Source: Virol J. 2008 Mar 27;5:47. doi: 10.1186/1743-422X-5-47 (PMC2289809; doi:10.1186/1743-422X-5-47)
Supplement: Additional file 1 — Table. Review of case reports of severe CMV infections in immunocompetent patients. Data extracted from studies that were not included in previous relevant reviews, regarding cases of severe CMV infections in immunocompetent patients. [file 1743-422X-5-47-S1.doc]

# Additional file 1.

# Table. Review of case reports of severe CMV infections in immunocompetent patients.

| Reference/ **Year of publication** | **Age/sex** | **Site involved** | **Comorbidity** | **Method of diagnosis** | **Antiviral therapy** | **Other therapy** | **Outcome** |
| --- | --- | --- | --- | --- | --- | --- | --- |
| **Gastrointestinal tract** | | | | | | | |
| [1]/  2007 | 71/M | Proctitis | Esophageal adenocarcinoma, diabetes mellitus, 2^nd^-degree heart block | Biopsy | None | Proctectomy | Death |
| [2]/  2007 | 62/F | Terminal ileitis | Goblet cell carcinoid tumour at the appendix | Serology  PCR of blood  Biopsy | Ganciclovir | Ileocaecal resection  Antibiotics | Good |
| [3]/  2006 | 87/M | Colitis | None | Serology | Ganciclovir | None | Good |
| [4]/  2006 | 76/F | Colitis | Diabetes mellitus  Hypertension  Mastectomy 20 years ago | Biopsy  Serology  CMV antigen in blood samples | Ganciclovir  +Valganciclovir | None | Good |
| [5]/  2006 | 64/F | Colitis | Cerebrovascular disease  Hypertension  Arrhythmia | Biopsy  PCR | Ganciclovir +Valganciclovir | Antibiotics  Inotropic treatment  Heparin, warfarin | Good |
| [6]/  2006 | 1 /M | Chronic active gastroduodenitis, complicated by pancreatitis and acute peritonitis | None | Serology  PCR in frozen stool and urine  Biopsy | None | Steroids  Drainage operation for peritonitis | Good |
| [7]/  2006 | 3-months/M | Duodenitis  Haemolytic anaemia | None | Serology  Urine, blood, and saliva culture  Biopsy | Vanganciclovir | IV antibiotics  RBCs tranfusion | Good |
| [8]/  2005 | 80/M | Colitis, thrombocytopenia | Peripheral facial paralysis | PCR in blood  Serology | None | Steroids  Immunoglobulins | Good |
| [9]/  2005 | 82/M | Colitis | ND | Biopsy | Valganciclovir | Antibiotics  Mesalamine | Good |
| [10]/  2004 | 60/F | Enteritis | Diabetes mellitus | Biopsy  Serology | Ganciclovir  Antibiotics | Enterectomy | Good |
| [11]/  2004 | 2month/M | Enterocolitis | None | Urine cultures | None | Fresh frozen plasma, Antibiotics | Good |
| [12]/  2004 | 22/M | Colitis | Rubella | Serology | None | None | Good |
| [13]/  2004 | 29/M | Colitis | Preceding amebiasis | Serology  Biopsy | None | Antibiotics for amebic colitis | Good |
| [14]/  2004 | 50/F | Proctitis | Crohn’s disease | Serology serum  Biopsy | Ganciclovir Valganciclovir | Partial colectomy  Mesalamine | Good |
| [15]/  2003 | 22/F | Pancolitis | Ulcerative colitis  Primary sclerosing cholangitis | Serology  PP65 CMV  Biopsy | Ganciclovir | Mesalazine  Steroids  Antibiotics | Good |
| [16]/  2003 | 59/F | Colitis  Haemolytic anaemia | Diabetes mellitus 2  Hypertension  G6PD deficiency | Serology  Biopsy | Ganciclovir | Antibiotics | Good |
| [17]/  2002 | 62/M | Colitis | Ulcerative colitis | Biopsy  Serology serum | Ganciclovir | Mesalamine, 5 amino-salicylic acid  Steroids  Antibiotics  Subtotal colectomy | Good |
| [18]/  2002 | 57/F | Enterocolitis | Appendectomy  Uterine myoma | Biopsy  Serology | None | None | Cood |
|  | 18/F | Enteritis | None | Biopsy (DNA analysis) | None | Steroids | Good |
|  | 43/M | Enteritis | None | Biopsy | None | Enterectomy | Good |
|  | 68/F | Enteritis | None | Biopsy | None | Enterectomy | Good |
|  | 35/M | Enteritis | None | Biopsy | None | None | Good |
| [19]/  2001 | 57/M | Small intestine ulcers/ Bleeding | None | Biopsy | None | Transfusion of RBCs  Resection of the proximal ileum | Good |
| [20]/  2001 | 57/F | Colitis | Acute renal insufficiency,diabetes mellitus 2, coronary artery disease, dyslipidaemia, obesity, hypothyroidism. | Biopsy | Ganciclovir | Partial colectomy, ileostomy | Good |
| [21]/  2000 | 68/M | Colitis | None | Biopsy | Ganciclovir | IV Antibiotics  Colectomy | Death (due to post-operative complications) |
| [22]/  2000 | 76/M | Pancolitis | Ulcerative colitis | Biopsy  Culture  PCR | Ganciclovir | None | Good |
| [23]/  1999 | 5-week/M | Enterocolitis | None | Biopsy  CMV early antigen (in urine)  Urine culture | Ganciclovir | Antibiotics  IV IG | Good |
| [24]/  1998 | 38/F | Exacerbation of silent ulcerative colitis | Ulcerative colitis | Serology  PCR in the urine | None | 5-aminosalicylic acid | Good |
|  | 20/M | CMV colitis, coincidence with the onset of IBD | Bronchial asthma  Congenital spherocytosis (Splenectomy at the age of 12 years) | Serology | None | Metronidazole, 5-aminosalicylic acid | Good |
| [25]/  1998 | 29/F | Pouchitis | Ulcerative colitis | Biopsy  Serology  Urine-pouch tissue culture | Ganciclovir | Antibiotics Mesalamine | Good |
| [26]/  1998 | 23/M | Gastroenteritis | None | Serology  Urine culture | None | Omeprazole | Good |
| [27]/  1997 | 5 week/M | Colitis | Cow’s milk allergy (simultaneous diagnosis) | Biopsy  Serology  Throat/urine culture | None | Hypoallergenic feeding formula | Good |
| Hematologic and immunologic disorders | | | | | | | |
| [28]/  2005 | 33/M | Profound thrombocytopenia | None | Serology | None | Platelet transfusion  Methylprednisolone, g-immunoglobulin | Good |
| [29]/  2004 | 38/M | Haemolytic anaemia | None | Serology | None | None | Good |
| [30]/  2004 | 32/F | Thrombocytopenia  SIADH | Pandysautonomia | Serology | None | Prednisolone | Good |
| [31]/  2003 | 38/M | Severe thrombocytopenia | No | Serology | None | IV immunoglobulins  Steroids | Good |
| [32]/  2002 | 62 /M | Monoclonal gammopathy | Hypertension | Serology | None | Antibiotics | Good |
| [33]/  2002 | 33/F | Thrombocytopenic purpura | None | Serology  PCR | None | Steroids | Good |
| [34]/  2000 | ND | Leg purpura (3)  Splenic haematoma (2) | ND | Serology | ND | Splenectomy in two | Good |
| [35]/  1997 | 41/M | Systemic purpura, epistaxis  Myelodysplasia | None | Serology  Blood PCR | None | Prednizolone  Platelet transfusions | Good |
| [36]/  1994 | 25/F | Spleen rupture | ND | Serology  Spleen biopsy | None | Splenectomy | Good |
| [37]/  1992 | 33/M | Severe thrombocytopenia/ purpura | None | Serology | None | Prednisone | Good |
| [38]/  1986 | ND | Haemolysis | ND | ND | ND | ND | ND |
|  | ND | Haemolysis | ND | ND | ND | ND | ND |
|  | ND | Haemolysis | ND | ND | ND | ND | ND |
|  | ND | Ruptured spleen | ND | ND | ND | ND | ND |
| Thrombosis |  |  |  |  |  |  |  |
| [39]/  2007 | 79/F | Pulmonary embolism | Rhizomelic pseudopolyarthritis | PP65 antigenemia  Serology  Blood culture | None | Anticoagulant treatment  Corticosteroids | Good |
| [40]/  2006 | 63/F | DIC | None | Serology | None | Plasma exchange  Blood and platelet transfusion | Good |
| [41]/  2005 | 33/M | PVT | Factor V mutation heterozygote  Femoral vein thrombosis 10 y ago | Serology  PP65 antigenemia | None | Anticoagulant treatment | Good |
| [42]/  2003 | 35/M | Multiple pulmonary emboli | None | Serology | None | LMWH  Warfarin | Good |
| Liver/Gallbladder | | | | | | | |
| [43]/  2006 | 36/M | Hepatitis (and Myopericarditis) | None | Serology | None | Salicylic acid | Good |
| [44]/  2006 | 34/M | Hepatitis, Portal Hypertension,  pulmonary involvement | None | Serology serum | None | Supportive treatment only | Good |
| [45]/  2006 | 36/M | Cholestatic hepatitis  (and Spleen granuloma) | Splenectomy transfusion | Serology  PP65 antigenemia,  PCR (in blood and spleen tissue) | Ganciclovir | ND | Good |
| [46]/  2006 | 47/F | Hepatitis, PVT | Oral contraceptives | Serology | None | Heparin  Antivitamins K | Good |
| [47]/  2005 | 55/M | Cholangiitis, pancreatitis | Diabetes mellitus  Hypertension | Biopsy  Serology | None | Operation | Good |
| [48]/  2005 | 72/F | Papillitis | Distal cholangiocarcinoma | Biopsy | None | Operation | Good |
| [49]/  2004 | 17month/F | Hepatitis | None | PP65 antigenemia  CMV early-antigen in urine  PCR of plasma  Liver biopsy | Ganciclovir | IV Immunoglobulins and aspirin | Good |
| [50]/  2001 | 8month/M | Hepatitis | None | PCR in blood , ISH of liver | Ganciclovir | HGG | Good |
|  | 1month/F | Hepatitis | Hypertrophic pyloric stenosis | Serology , blood and urine cultures  ISH of liver | Ganciclovir | HGG | Good |
|  | 2 month/M | Hepatitis | None | Serology , blood and urine cultures  ISH of liver | No | HGG | Good |
| [51]/  1999 | 34/M | Acute cholestatic hepatitis | None | PP65 antigenemia Serology  Biopsy | Ganciclovir | ND | Good |
| [52]/  1998 | 32/F | Hepatitis | Pregnancy | Blood culture  Serology  Liver biopsy | Ganciclovir | Supportive treatment | Good |
| Central nervous system | | | | | | | |
| [53]/  2007 | 45/F | Meningitis | Hypertension | PCR | Ganciclovir | None | Good |
| [54]/  2007 | 34/M | Neuromyelitis optica,  rhabdomyolysis | None | Serology | Valganciclovir | Antibiotics  Steroids  Mycophenolate mofetil | Good |
| [55]/  2006 | 64/F | Myelitis | None | Serology | Ganciclovir | Corticosteroids | Partial improvement |
| [56]/  2006 | 51/M | Encephaloradiculomyelitis, coinfection with HHV-7 | Νone | PCR of CSF  Serology | Ganciclovir | Dexamethasone Penicillin  Chemicetin | Partial improvement |
| [57]/  2005 | 73/M | Acute transverse myelitis | None | Serology in CSF  Serum serology | Ganciclovir Valganciclovir | Steroids | Partial improvement |
| [58]/  2003 | 31/M | Acute transverse myelitis | None | Serology  PP65 antigenemia | Ganciclovir | Methylprednisolone | Partial improvement |
| [59]/  2003 | 1  Relapsing illness 15 | IUGR/Deafness  Relapsing illness, mean duration of symptoms 7.8 weeks | ND | ND | ND | ND |  |
| [60]/  2002 | 16/M | Transverse myelitis | None | PCR of blood  Serology serum | IV acyclovir | IV IG  Steroids | Partial improvement |
| [61]/  2001 | 54/M | Myelitis | ND | PCR of CSF | ND | ND | ND |
|  | 21 /F | Myelitis | Pregnancy | PCR of CSF | ND | ND | ND |
|  | 25/M | Myeloradiculitis | ND | PCR of CSF | ND | ND | ND |
| [62]/  1999 | 54/M | Transverse myelitis | None | Serology, blood culture  PCR of blood pp65 antigenemia | Ganciclovir | None | Good |
| [63]/  1998 | ND | Encephalitis | ND | ND | ND | ND | ND |
| [64]/  1997 | 36/M | Myeloradiculopathy | None | Serology in serum  Serology in CSF | None | Analgesics  NSAIDS | Good |
| [65]/  1995 | 24/F | Transverse myelitis | None | Serology | None | Steroids  Ventilatory support | Good |
| [66]/  1995 | 28/F | Cranial nerve III palsy | Pregnancy | Serology serum  Culture of amniotic fluid | None | Therapeutic termination of pregnancy | Good |
| [67]/  1994 | 7/M | Meningoencephalitis | Arachnoidal cyst in the posterior cranial fossa | PCR in CSF  Serology | ND | ND | Good |
|  | 6/M | Meningoencephalitis | Arachnoidal cyst in the posterior cranial fossa | PCR in CSF  Serology | ND | ND | Good |
| [36]/  1994 | 23/F | Meningoencephalitis | ND | Serology | Acyclovir | ND | Good |
| Ophthalmologic involvement | | | | | | | |
| [68]/  2007 | 77/M | Corneal endotheliitis | Intractable corneal oedema | PCR of aqueous humour  Confocal microscopy | Ganciclovir topical and IV | Acyclovir  Steroids | Good |
| [69]/  2007 | 77/M | Retinitis | Diabetes mellitus 2, bilateral pseudophakia, reccurent retinal detachment in the left eye | Slit-lamp examination, PCR in the aqueous humour, serology | Intravitreal injection of ganciclovir  IV ganciclovir and valganciclovir | Surgeries, intravitreal injection of triamcinolone and photodynamic therapy | Failure |
|  | 69/M | Retinitis | Diabetes mellitus 2, central retinal vein occlusion in the right eye | PCR in the aqueous humour, serology | Intravitreal injection of ganciclovir, valganciclovir | Insulin therapy, intravitreal injection of triamcinolone acetonide, cataract surgery | Good |
| [70]/  2007 | 55/F | Unilateral anterior uveitis | ND | PCR of aqueous humour  Serology | Valganciclovir | Steroids  Mydriatics  Antiglaucoma treatment | Good  Recurrence after 1 year |
|  | 45/M | Unilateral anterior uveitis | ND | PCR of aqueous humour  Serology | Valganciclovir | Steroids  Antiglaucoma treatment | Good  Recurrence after 3 months |
|  | 22/M | Unilateral anterior uveitis (secondary cataract and glaucoma) | Intermittent granulomatous anterior uveitis since age 11 | PCR | None | Trabeculectomy  Cataract extraction  Implantation of drainage device | Failure |
| [71]/  2006 | 80/M | Anterior unilateral uveitis  Secondary glaucoma | ND | Serology | Ganciclovir iv or foscarnet iv, followed by valganciclovir po | Rimexolone,dorzolamide,brimonidine | Good after prolonged antiviral treatment (2 relapses when treatment initially discontinued) |
|  | 59/M | Anterior unilateral uveitis  Secondary glaucoma | N.D | Serology and/or PCR of aqueous humour | Ganciclovir iv or foscarnet iv, followed by valganciclovir po | Rimexolone, timolol, apraclonidine | Good without relapse |
|  | 45/F | Anterior unilateral uveitis  Secondary glaucoma | N.D | Serology and/or PCR of aqueous humour | Ganciclovir iv or foscarnet iv, followed by valganciclovir po | Rimexolone, timolol, apraclonidine, brinzolamide | Good after prolonged antiviral treatment (1 relapses when treatment initially discontinued) |
|  | 30/M | Anterior unilateral uveitis  Secondary glaucoma | N.D | Serology and/or PCR of aqueous humour | Ganciclovir iv or foscarnet iv, followed by valganciclovir po | Rimexolone, timolol, apraclonidine | Good without relapse |
|  | 42/F | Anterior unilateral uveitis  Secondary glaucoma | N.D | Serology and/or PCR of aqueous humour | Oral valganciclovir | dorzolamide | Good after prolonged antiviral treatment (2 relapses when treatment initially discontinued) |
| [72]/  2005 | 75/M | Retinitis | Diabetes mellitus 2 | PCR of the vitreous | Valganciclovir | IV Triamcinolone  Vancomycin Ceftazidime  Steroids | Good |
| [73]/  2004 | 6-week/F | Retinitis and Protein-losing gastroenteropathy | None | Serology  PP65 antigenemia  PCR (blood, urine, mother’s breast milk) | Ganciclovir | γ-globulin | Good |
| [74]/  2002 | 66/M | Anterior uveitis with sectoral iris atrophy (recurrences) | ND | Serology  PCR of aqueous humour | Acyclovir (initially)  Ganciclovir | Steroids  Timolol  Cyclopentolate | Good |
|  | 36/M | Anterior uveitis with sectoral iris atrophy (recurrence) | Myocardial infarction  Recurrent aphthous ulcers associated with CMV | PCR of aqueous humour  Serology | Acyclovir (initially)  Ganciclovir | Steroids  Timolol  Atropine | Marked clinical improvement |
| [75]/  1996 | 32/F | Bilateral papillitis | None | Serology  Early antigen in urine | Foscarnet | Steroids  Acyclovir | Good |
| Skin | | | | | | | |
| [76]/  2006 | 12 Weeks/M | Perineal ulcers | Supraventricular tachycardia  Abdominales ascites  Pericardial effusion | Biopsy (immunoperoxidase stain)  Serology | None | Zinc oxide  Steroids | Good |
| [77]/  2003 | 47/F | Cutaneous eruption | None | Serology  PCR of biopsy specimens | None | None | Good |
| [33]/  2002 | 40/F | Vasculitic Rash | None | Serology | None | Steroids | Good |
| [34]/  2002 | ND | Vasculitic Rash | ND | ND | ND | ND | Good |
| **Lung involvement** | | | | | | | |
| [78]/  2005 | 49/F | Septal capillary injury syndrome | Autoimmune diathesis | Serology  PCR of blood  ISH in skin biopsy cells | Antiviral therapy | Immunosuppressive therapy | Partial improvement (reccurrence) |
|  | 54/F | Septal capillary injury syndrome | Autoimmune diathesis | Serology  Post-mortem lung tissue viral culture  ISH in autopsy cells | None | None | Death |
|  | 55/M | Septal capillary injury syndrome | Autoimmune diathesis | Serology  BAL rapid CMV antigen test  ISH | Antiviral therapy | Immunosuppressive therapy | Partial improvement (reoccurrence) |
|  | 64/F | Septal capillary injury syndrome | Autoimmune diathesis | Serology  Blood, urine culture; BAL  ISH | Antiviral therapy | Immunosuppressive therapy | Good |
| [79]/  2004 | 71/F | Usual interstitial pneumonia | Acute myocardial infarction, Diabetes mellitus, Arterial hypertension | Open lung biopsy  Immunohistochemistry, PCR | Ganciclovir | PTCA | Death |
| [80]/  2003 | 47/M | Pneumonia | None | Transbronchial biopsy, thoracotomy-excision of the mass-Culture | None | ND | Good |
| [63]/  1998 | ND | Interstitial Pneumonitis | ND | ND | Ganciclovir | None | Good |
| [81]/  1997 | 32/F | Interstitial pneumonitis | None | Serology  Complement fixation test | Ganciclovir | Antibiotics | Good |
|  | 44/M | Pneumonitis | None | Serology  Complement fixation test | Ganciclovir | Antibiotics | Good |
| **Significant weight loss** | | | | | | | |
| [82]/  2004 | 66/M | 25-Kg weight loss | Hepatosplenomegaly | Serology  Blood culture | None | None | Good |
|  | 32/M | 13-Kg weight loss | Splenomegaly | Serology  Urine culture | None | None | Good |

**Abbreviations** M : male, F :female, IV: intravenous administration, IG: immunoglobulin, DIC: disseminated intravascular coagulation, PVT: portal vein thrombosis, LMWH: low molecular wight heparin, PCR: polymerase chain reaction, TPA: tissue plasminogen activator, SIADH: syndrome of inappropriate secretion of antidiuretic hormone, G-CSF: granulocyte-colony stimulating factor, HGG: hyperimmune gammaglobulin, HHV-7: human herpes-virus 7, IBD: inflammatory bowel disease, ACV: acyclovir, TTP: thrombotic thrombocytopenic purpura, CSF : cerebrospinal fluid, IUGR : intrauterine growth retardation, ND : no data

**References**

1. Alam I, Shanoon D, Alhamdani A, Boyd A, Griffiths AP, Baxter JN. Severe proctitis, perforation, and fatal rectal bleeding secondary to cytomegalovirus in an immunocompetent patient: report of a case. Surg Today 2007; 37:66-9.
2. Ng SC, Noursadeghi M, von Herbay A, Vaizey C, Pitcher MC, Flanagan KL. Cytomegalovirus ileitis associated with goblet cell carcinoid tumour of the appendix. J Infect 2007;54:e153-6.
3. Fleischer B, Morgenthaler J, Tippel G, Kuhfus A, Gieseler U, Stolte M. Cytomegalovirus-induced colitis in an immunocompetent old patien]. Med. Klin. (Munich). 2006;101:835-8.
4. Carter D, Olchovsky D, Pokroy R, Ezra D. Cytomegalovirus-associated colitis causing diarrhea in an immunocompetent patient. World J Gastroenterol 2006;12:6898-9.
5. Lockwood MR, Liddle J, Kitsanta P. Cytomegalovirus colitis--an unusual cause for diarrhoea in an elderly woman. Age Ageing 2006;35:198-200.
6. Shimizu M, Ohta K, Wada H, Sumita R, Yachie A, Koizumi S. Cytomegalovirus-associated protracted diarrhoea in an immunocompetent boy. J Paediatr Child Health 2006;42:259-62.
7. Buonuomo PS, Maurizi P, Valentini P, Mastrangelo S, Lazzareschi I, Ridola V, et al. Successful treatment with oral valganciclovir in immunocompetent infant with gastrointestinal manifestations of cytomegalovirus infection. J Perinatol 2006;26:648-9.
8. Alliot C, Barrios M. Cytomegalovirus-induced thrombocytopenia in an immunocompetent adult effectively treated with intravenous immunoglobulin: a case report and review. Hematology 2005;10:277-9.
9. Siegal DS, Hamid N, Cunha. Cytomegalovirus colitis mimicking ischemic colitis in an immunocompetent host. BA Heart Lung. 2005;34:291-4.
10. Petrogiannopoulos CL, Kalogeropoulos SG, Dandakis DC, Hartzoulakis GA, Karahalios GN, Flevaris CP, et al. Cytomegalovirus enteritis in an immunocompetent host. Chemotherapy 2004;50:276-8.
11. Hinds R, Brueton MJ, Francis N, Fell JM. Another cause of bloody diarrhoea in infancy: cytomegalovirus colitis in an immunocompetent child. J Paediatr Child Health 2004;40:581-2.
12. Sugisaki K, Maekawa S, Mori K, Ichii O, Kanda K, Tai M, et al. H. Self-limited colitis during the course of rubella and cytomegalovirus infection in an immunocompetent adult. Intern Med 2004;43:404-9.
13. Lee CS, Low AH, Ender PT, Bodenheimer HC. Cytomegalovirus colitis in an immunocompetent patient with amebiasis: case report and review of the literature. Mt Sinai J Med 2004;71:347-50.
14. Filizzola MJ, Shazia G, Martinez F, Rauf SJ. Cytomegalovirus infection exacerbating Inflammatory Bowel Disease. Infect Med 2004;21:567-570
15. Streetz KL, Buhr T, Wedemeyer H, Bleck J, Schedel I, Manns MP, et al.Acute CMV-colitis in a patient with a history of ulcerative colitis. Scand J Gastroenterol 2003;38:119-22.
16. Farah R, Sbeit W, Nassar F, Cohen H, Reshef R. Cytomegalovirus colitis and haemolytic anaemia in a glucose-6-phosphate dehydrogenase-deficient immunocompetent patient. Eur J Gastroenterol Hepatol. 2003;15:1029-31.
17. Malhi NS, Bhasin DK, Gupta NM, Vaiphei K, Singh K. Exacerbation of ulcerative colitis by cytomegalovirus infection in an immunocompetent Indian patient. Trop Gastroenterol. 2002;23:88-90.
18. Sakamoto I, Shirai T, Kamide T, Igarashi M, Koike J, Ito A, et al. Cytomegalovirus enterocolitis in an immunocompetent individual. J Clin Gastroenterol 2002;34:243-6.
19. Choi SW, Chung JP, Song YK, Park YN, Chu JK, Kim DJ, et al. Lower gastrointestinal bleeding due to cytomegalovirus ileal ulcers in an immunocompetent man. Yonsei Med J 2001;42:147-151.
20. Larkin JA, Li-Espino E. CMV Colitis in an Elderly Patient. Infect Med 2001;18:396-398
21. Rivera FM, Avalos E. A rare case of CMV colitis in an immunocompetent patient. Am J Gastr 2000;95:2601-2602
22. Kraus M, Meyenberger C, Suter W. Generalized intestinal CMV infection with protein-losing syndrome in ulcerative colitis. Schweiz Med Wochenschr 2000;130:1600-5.
23. Fox LM, Gerber MA, Penix L, Rizzi A, Hyams JS. Intractable diarrhea from Cytomegalovirus enterocolitis in an immunocompetent infant. Pediatrics 1999;103;e10
24. Rachima C, Maoz E, Apter S, Thaler M, Grossman E, Rosenthal T. Cytomegalovirus infection associated with ulcerative colitis in immunocompetent individuals. Postgrad Med J 1998; 74:486-9.
25. Moonka D, Furth EE, MacDermott RP, Lichtenstein GR. Pouchitis associated with primary cytomegalovirus infection. Am J Gastroenterol 1998;93:264-6.
26. Bencharif L, Cathebras P, Bouchou K, Gouilloud S, Fichtner C, Rousset H. Exudative gastroenteropathy revealing primary CMV infection in an immunocompetent adult] Rev Med Interne 1998; 19:288-90.
27. Jonkhoff-Slok TW, Veenhoven RH, de Graeff-Meeder ER, Büller HA. An immunocompetent infant with cow's milk allergy and cytomegalovirus colitis. Eur J Pediatr 1997;156:528-9.
28. Nomura K, Matsumoto Y, Kotoura Y, Shimizu D, Kamitsuji Y, Horiike S, et al. Thrombocytopenia due to cytomegalovirus infection in an immunocompetent adult. Hematology 2005;10:405-6.
29. Veldhuis W, Janssen M, Kortlandt W, van Houte A, van de Ree M. Coombs-negative severe haemolytic anaemia in an immunocompetent adult following cytomegalovirus infection. Eur J Clin Microbiol Infect Dis 2004;23:844-7.
30. Sato H, Kamoi K, Saeki T, Yamazaki H, Koike T, Miyamura S, et al.Syndrome of inappropriate secretion of antidiuretic hormone and thrombocytopenia caused by cytomegalovirus infection in a young immunocompetent woman. Intern Med 2004;43:1177-82.
31. Ichiche M, Fontaine C, Lacor P. Severe thrombocytopenia secondary to cytomegalovirus infection in an immunocompetent adult. Eur J Intern Med 2003;14:56-59
32. Bühler S, Laitinen K, Holthöfer H, Järvinen A, Schauman KO, Hedman K. High rate of monoclonal gammopathy among immunocompetent subjects with primary cytomegalovirus infection. Clin Infect Dis 2002;35:1430-3.
33. Crowley B, Dempsey J, Olujohungbe A, Khan A, Mutton K, Hart CA. Unusual manifestations of primary cytomegalovirus infection in patients without HIV infection and without organ transplants. J Med Virol 2002;68:237-40.
34. Bonnet F, Morlat P, Neau D, Viallard JF, JM, Dupon M, et al.Hematologic and immunologic manifestations of primary cytomegalovirus infections in non-immunocompromised hospitalized adults Rev Med Interne 2000;21:586-94
35. Miyahara M, Shimamoto Y, Yamada H, Shibata K, Matsuzaki M, Ono K. Cytomegalovirus-associated myelodysplasia and thrombocytopenia in an immunocompetent adult. Ann Hematol 1997;74:99-101.
36. Ragnaud JM, Morlat P, Gin H, Dupon M, Delafaye C, du Pasquier P, et al.[Clinical, biological and developmental aspects of cytomegalovirus infection in immunocompetent patients: apropos of 34 hospitalized patients]. Rev Med Interne 1994;15:13-8.
37. Wright JG. Severe thrombocytopenia secondary to asymptomatic cytomegalovirus infection in an immunocompetent host. J Clin Pathol 1992;45:1037-8.
38. Horwitz CA , Henle W, Henle G, Snover D, Rudnick H, Balfour HH Jr et al. Clinical and laboratory evaluation of cytomegalovirus-induced mononucleosis in previously healthy individuals.Report of 82 cases. Medicine (Baltimore). 1986;65(2):124-34.
39. Delbos V, Abgueguen P, Chennebault JM, Fanello S, Pichard E. Acute cytomegalovirus infection and venous thrombosis: role of antiphospholipid antibodies. J Infect 2007;54:e47-50.
40. Niewold TB, Bundrick JB. Disseminated intravascular coagulation due to cytomegalovirus infection in an immunocompetent adult treated with plasma exchange. Am J Hematol 2006 ;81:454-7.
41. Rovery C, Granel B, Parola P, Foucault C, Brouqui P. Acute cytomegalovirus infection complicated by venous thrombosis: a case report. Ann Clin Microbiol Antimicrob 2005;124:11.
42. Youd P, Main J, Jackson E. Cytomegalovirus infection and thrombosis: a causative association? J Infect 2003;46:141-2.
43. Zubiaurre L, Zapata E, Bujanda L, Castillo M, Oyarzabal I, Gutiérrez-Stampa MA, et al.Cytomegalovirus hepatitis and myopericarditis. World J Gastroenterol 2007;13:647-8.
44. Tzavella K, Zantidis A, Economou I, Mandraveli K, Alexiou-Daniel S, Dimitriadis A, et al.Portal hypertension caused by acute cytomegalovirus infection with liver involvement in an immunocompetent patient. Scand J Infect Dis 2007;39:177-8.
45. Assy N, Gefen H, Schlesinger S, Karim W. Reactivation versus Primary CMV Infection after Splenectomy in Immunocompetent Patients. Dig Dis Sci 2007;52:3477-9.
46. Girszyn N, Leport J, Baux N, Kahn JE, Blétry O. [Portal vein thrombosis associated with acute cytomegalovirus infection in an immunocompetent patient]. Rev Med Interne 2006;27:426-8.
47. Oku T, Maeda M, Waga E, Wada Y, Nagamachi Y, Fujita M, et al.Cytomegalovirus cholangitis and pancreatitis in an immunocompetent patient. J Gastroenterol 2005;40:987-92.
48. Ruiz-Tovar J, Martín-Pérez E, Gamallo-Amat C. Distal cholangiocarcinoma associated with papillitis with viral CMV inclusions. Dig Surg 2005;22:464-6.
49. Hadaya K, Kaiser L, Rubbia-Brandt L, Gervaix A, Diana A. Ganciclovir for severe cytomegalovirus primary infection in an immunocompetent child. Eur J Clin Microbiol Infect Dis 2004;23:218-20.
50. Tajiri H, Kozaiwa K, Tanaka-Taya K, Tada K, Takeshima T, Yamanishi K, et al. Cytomegalovirus hepatitis confirmed by in situ hybridization in 3 immunocompetent infants. Scand J Infect Dis 2001;33:790-3.
51. Serna-Higuera C, González-García M, Milicua JM, Muñoz V. Acute cholestatic hepatitis by cytomegalovirus in an immunocompetent patient resolved with ganciclovir. J Clin Gastroenterol 1999;29:276-7.
52. Miguelez M, Gonzalez A, Perez F. Severe cytomegalovirus hepatitis in a pregnant woman treated with ganciclovir. Scand J Infect Dis 1998;30:304-5.
53. Rafailidis PI, Kapaskelis A, Falagas ME. Cytomegalovirus meningitis in an immunocompetent patient. Med Sci Monit. 2007;13:CS107-109.
54. Tran C, Du Pasquier RA, Cavassini M, Guex-Crosier Y, Meuli R, Ciuffreda D, et al.Neuromyelitis optica following CMV primo-infection. J Intern Med 2007; 261:500-3.
55. Ben Abdelhafidh N, Battikh R, Laabidi J, M'sadek F, Ajili F, Ben Moussa M, et al. [Cytomegalovirus myelitis in immunocompetent adult]. Rev Med Interne 2006;27:883-5
56. Ginanneschi F, Donati D, Moschettini D, Dominici F, Cermelli C, Rossi A. Encephaloradiculomyelitis associated to HHV-7 and CMV co-infection in immunocompetent host. Clin Neurol Neurosurg 2007; 109:272-6.
57. Rigamonti A, Usai S, Ciusani E, Bussone G. Atypical transverse myelitis due to cytomegalovirus in an immunocompetent patient. Neurol Sci; 26:351-4.
58. Fux CA, Pfister S, Nohl F, Zimmerli S. Cytomegalovirus-associated acute transverse myelitis in immunocompetent adults. Clin Microbiol Infect 2003; 9:1187-90.
59. Wreghitt TG, Teare EL, Sule O, Devi R, Rice P. Cytomegalovirus infection in immunocompetent patients. Clin Infect Dis. 2003;37:1603-6.
60. Karacostas D, Christodoulou C, Drevelengas A, Paschalidou M, Ioannides P, Constantinou A, et al.Cytomegalovirus-associated transverse myelitis in a non-immunocompromised patient. Spinal Cord. 2002; 40:145-9.
61. Kleinschmidt-DeMasters BK, Gilden DH. The expanding spectrum of herpesvirus infections of the nervous system. Brain Pathol 2001;11:440-51.
62. Giobbia M, Carniato A, Scotton PG, Marchiori GC, Vaglia A. Cytomegalovirus-associated transverse myelitis in a non-immunocompromised patient. Infection 1999; 27:228-30.
63. Faucher JF, Abraham B, Segondy M, Jonquet O, Reynes J, Janbon F. Acquired cytomegalovirus infections in immunocompetent adults: 116 cases. Presse Med 1998;27:1774-9.
64. Etaquil N, Benyahya E, Janani S, el Fatimi A, Bennis R, Mkinsi O. Diffuse arthralgia and myalgia as the first manifestation of benign myeloradiculopathy due to cytomegalovirus infection in an immunocompetent patient. Rev Rhum Engl Ed 1997;64:57-8.
65. Baig SM, Khan MA. Cytomegalovirus-associated transverse myelitis in a non-immunocompromised patient. J Neurol Sci 1995;134:210-1.
66. Tiab M, Raffi F, Hamidou M, Dupas B, Barrier JH. [Paralysis of the 3d cranial nerve disclosing primary cytomegalovirus infection in an immunocompetent adult] Ann Med Interne (Paris) 1995;146:130-2.
67. Darin N, Bergström T, Fast A, Kyllerman M. Clinical, serological and PCR evidence of cytomegalovirus infection in the central nervous system in infancy and childhood. Neuropediatrics. 1994;25:316-22.
68. Shiraishi A, Hara Y, Takahashi M, Oka N, Yamaguchi M, Suzuki T, et al.Demonstration of "owl's eye" morphology by confocal microscopy in a patient with presumed cytomegalovirus corneal endotheliitis. Am J Ophthalmol 2007;143:715-7
69. Delyfer MN, Rougier MB, Hubschman JP, Aouizérate F, Korobelnik JF. Cytomegalovirus retinitis following intravitreal injection of triamcinolone:report of two cases. Acta Ophthalmol Scand. 2007; 85(6):681-3.
70. van Boxtel LA, van der Lelij A, van der Meer J, Los LI. Cytomegalovirus as a Cause of Anterior Uveitis in Immunocompetent Patients. Ophthalmology 2007; 114(7):1358-62.
71. de Schryver I, Rozenberg F, Cassoux N, Michelson S, Kestelyn P, Lehoang P, et al.Diagnosis and treatment of cytomegalovirus iridocyclitis without retinal necrosis. Br J Ophthalmol 2006; 90:852-5.
72. Saidel MA, Berreen J, Margolis TP. Cytomegalovirus retinitis after intravitreous triamcinolone in an immunocompetent patient. Am J Ophthalmol 2005;140:1141-3.
73. Iwanaga M, Zaitsu M, Ishii E, Nishimura Y, Inada S, Yoshiki H, et al. Protein-losing gastroenteropathy and retinitis associated with cytomegalovirus infection in an immunocompetent infant: a case report. Eur J Pediatr 2004;163:81-4.
74. Markomichelakis NN, Canakis C, Zafirakis P, Marakis T, Mallias I, Theodossiadis G. Cytomegalovirus as a cause of anterior uveitis with sectoral iris atrophy. Ophthalmology 2002;109:879-82.
75. Baglivo E, Leuenberger PM, Krause KH. Presumed bilateral cytomegalovirus-induced optic neuropathy in an immunocompetent person. A case report. J Neuroophthalmol 1996;16:14-7.
76. Hancox JG, Shetty AK, Sangueza OP, Yosipovitch G. Perineal ulcers in an infant: an unusual presentation of postnatal cytomegalovirus infection. J Am Acad Dermatol 2006;54:536-9.
77. Oskay T, Karademir A, Kutluay L. Vesicular and pustular eruption related to cytomegalovirus in an immunocompetent patient. Clin Exp Dermatol 2003;28:610-2.
78. Magro C, Ali N, Williams JD, Allen JN, Ross P. Cytomegalovirus-associated pulmonary septal capillary injury sine inclusion body change: a distinctive cause of occult or macroscopic pulmonary hemorrhage in the immunocompetent host. Appl. Immunohistochem. Mol Morphol 2005; 13:268-72.
79. Rizos M, Falagas ME, Tsiodras S, Betsou A, Foukas P, Michalopoulos A. Usual interstitial pneumonia associated with cytomegalovirus infection after percutaneous transluminal coronary angioplasty. Eur J Clin Microbiol Infect Dis. 2004;23:848-50.
80. Karakelides H, Aubry MC, Ryu JH. Cytomegalovirus pneumonia mimicking lung cancer in an immunocompetent host. Mayo Clin Proc. 2003;78:488-90.
81. Laing RB, Dykhuizen RS, Smith CC, Molyneaux PJ. Parenteral ganciclovir treatment of acute CMV infection in the immunocompetent host. Infection 1997;25:44-6.
82. Rodríguez-Baño J, Muniain MA, Borobio MV, Corral JL, Ramírez E, Perea EJ, et al. Cytomegalovirus mononucleosis as a cause of prolonged fever and prominent weight loss in immunocompetent adults. Clin Microbiol Infect 2004;10:468-70.
